# Knowledge, perceptions and behaviours regarding dietary management of adults living with phenylketonuria

**DOI:** 10.1111/jhn.13015

**Published:** 2022-04-27

**Authors:** Sarah J. Firman, Radha Ramachandran, Kevin Whelan

**Affiliations:** ^1^ Department of Nutritional Sciences King's College London London UK; ^2^ Department of Nutrition and Dietetics Guy's and St Thomas' NHS Foundation Trust London UK; ^3^ Adult Inherited Metabolic Diseases Guy's and St Thomas' NHS Foundation Trust London UK

**Keywords:** adults, dietary behaviours, dietary management, knowledge, perceptions, phenylketonuria

## Abstract

**Background:**

Lifelong dietary treatment remains the mainstay for many with phenylketonuria (PKU); however, adherence is known to reduce with age. It remains unclear whether knowledge and perceptions of the PKU diet amongst adults with PKU influence dietary behaviours.

**Methods:**

A nationwide questionnaire survey was performed to investigate the knowledge and perceptions, and associated diet behaviours of adults with PKU in the UK. The survey was sent to adults with PKU under the care of the host hospital and members of the National Society of PKU.

**Results:**

One hundred and thirty‐seven respondents (*n* = 78 females, 56.9%) completed the survey with a mean age of 34 years and 4 months (16–65 years). Sixty (43.8%) respondents had always followed a PKU diet, 39 (28.5%) returned to diet and 35 (25.5%) were off diet. Overall mean ± SD knowledge score was 75.2% ± 13.4%, with significantly higher scores for knowledge of PKU (80.7% ± 16.2%) compared to knowledge specifically of the PKU diet (72.6% ± 14.5%, *p* < 0.001). Knowledge was associated with dietary adherence. Respondents who always followed a PKU diet had similar knowledge to those who returned to diet, whereas respondents off diet had significantly lower scores. Perception of the diet was not a predictor of dietary adherence, with the exception of whether patients had concerns for their long‐term health when on diet or felt well when not following a diet.

**Conclusions:**

The present study highlights the importance of ongoing dietetic input in building knowledge and skills for dietary management. Further research is needed to understand the motivators and beliefs that influence dietary adherence.

## Key points

- Lifelong dietary treatment remains the mainstay for many with phenylketonuria (PKU); however, adherence is known to reduce with age.

- The present study aimed to determine whether knowledge and perceptions of the PKU diet amongst adults with PKU influence dietary behaviours.

- Knowledge of PKU and the PKU diet was associated with dietary adherence, highlighting the importance of ongoing dietetic input in building knowledge and skills for dietary management.

- Perception of the diet was not a predictor of dietary adherence.

- Further research is needed to understand the motivators and beliefs that influence dietary adherence.

## Graphical abstract

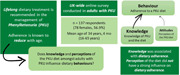

## INTRODUCTION

Phenylketonuria (PKU; OMIM 261600) is a rare inherited metabolic disorder of protein metabolism with a prevalence of 1:10,000 births in Europe. Dietary management involves the restriction of phenylalanine through a low protein diet, supplemented with specialist phenylalanine free or low phenylalanine protein substitutes providing all remaining amino acids and specialist low protein foods to ensure adequate energy and variety in the diet. All these dietary components are essential to the management of PKU. However, many find the diet challenging, and adherence has been shown to reduce with age, as revealed in a large UK‐based survey where only 57% of adults with PKU reported following a low phenylalanine diet.^1^ Current consensus advocates treatment for life^2^; therefore, understanding the factors that influence dietary behaviours and removing barriers to improve dietary adherence will improve patient outcomes.

The knowledge, attitude, behaviour theoretical model of health education suggests that behaviour (e.g., adherence to diet) is influenced by knowledge (e.g., knowledge of diet) and attitudes (e.g., perceptions of diet). Within PKU, studies have explored patients' and caregivers' knowledge of PKU and its dietary management, with some studies investigating the association of knowledge with adherence (behaviour) to the diet.^1^, ^3^, ^4^, ^5^, ^6^, ^7^, ^8^ However, evidence for knowledge as a predictor for metabolic control remains equivocal.

A questionnaire survey in 62 people with PKU and 161 caregivers, as well as another survey in 32 people with PKU, did not show associations between knowledge and adherence,^3^, ^4^ whereas a study in 29 patients and 16 caregivers reported no association between perceived knowledge and adherence, but found perceived barriers to treatment to impact phenylalanine levels.^7^ Moreover, a study in 218 people with PKU, including 45 aged ≥ 20 years, and 110 caregivers, found no correlation between knowledge and attitudes.^6^ By contrast, a survey in 46 caregivers and another in 144 caregivers of children with PKU reported their knowledge of PKU and its dietary management to correlate with their children's blood phenylalanine concentrations.^5^, ^8^ However, the findings only reached statistical significance for young children whose carers had higher knowledge scores^5^ and for knowledge specific to PKU exchanges.^8^ A study in 183 adults with PKU reported dietary behaviours, but provided no insight into whether behaviours were influenced by knowledge and attitudes.^9^ A common theme for all studies was the lack of comprehensive assessment of attitudes/perceptions of PKU dietary management, as well as a limited focus on the perceptions and knowledge specifically regarding the role of protein substitutes in the diet. Furthermore, majority of studies were conducted in caregivers, children, and/or younger adults with PKU, with no studies specially focussed on investigating the association of knowledge and perceptions on dietary adherence in adults with PKU.

Following a low phenylalanine diet requires an understanding of dietary sources of phenylalanine, determining protein content of foods and regular consumption of protein substitutes, all of which require consideration when investigating both knowledge and perceptions of the dietary management of PKU. Studies have investigated factors that influence adherence to the PKU diet^1^, ^6^, ^7^, ^10^, ^11^; however, to our knowledge, no studies to date have specifically investigated whether perceptions of the PKU diet are associated with dietary adherence.

It remains unclear whether knowledge and perceptions of both protein restriction and protein supplementation in the PKU diet amongst adults with PKU influence dietary behaviours. The present study aimed to investigate the knowledge and perceptions and behaviours of adults with PKU across the UK, regarding numerous dietary aspects of PKU management and the factors associated with these. This will help to inform the direction of clinical care and future research to optimise supporting people with PKU to follow a diet for life.

## METHODS

A nationwide questionnaire survey was performed regarding the knowledge and perceptions of dietary management, and diet behaviours of adults with PKU in the UK.

### Participants

Two approaches were adopted to optimise recruitment to increase representation and statistical power. First, all patients with PKU attending the Adult Inherited Metabolic Disorder Service at St Thomas' Hospital, London, were sent an email with information and an electronic link to the survey, and patients attending routine outpatient clinic appointments were also provided with the survey information. Second, to ensure opportunities for adults with PKU from across the UK to participate, the survey was advertised by the National Society of Phenylketonuria (NSPKU), shared with all members via email and newsletter and advertised on their patient organisation website and social media platforms. The PKU Research Patient Advisory Group at the host hospital aided the advertisement of the survey on social media.

The inclusion criteria were patients: ≥16 years of age; able to complete the online survey independently; currently residing in the UK, diagnosed with PKU on newborn screening; and having been on a PKU diet for a period of their life. Potential participants were excluded if they resided outside of the UK or were <16 years of age. Eligibility was determined by participant self‐report at the start of the questionnaire survey.

### Questionnaire design

This questionnaire consisted of four sections: (i) knowledge of PKU and the PKU diet; (ii) perception of the PKU diet; (iii) dietary behaviours and metabolic control; and (iv) demographics. The knowledge section consisted of 25 multichoice questions; eight on knowledge of PKU and 17 specific to PKU dietary management, including four questions adapted from a questionnaire by Bekhof *et al*.^3^ Each multiple‐choice knowledge question was scored as correct, incorrect or participants selected ‘don't know’. The perception section consisted of 25 attitude statements relating to the PKU diet, protein substitutes and the impact of the PKU diet on health and physical ability, developed by the research team, and was rated using a five‐point Likert scale (strongly agree to strongly disagree), in addition to two questions relating to their perceived importance of research on PKU and diet. The dietary behaviours and metabolic control section for respondents currently following a PKU diet, consisted of nine questions regarding current and historical dietary behaviours, including providing information on prescribed dietary regimen, current PKU exchanges, protein substitutes taken, frequency of phenylalanine monitoring, blood phenylalanine target and last recorded phenylalanine level. The dietary behaviours section for respondents who were currently off diet, consisted of three questions to determine the time since last on a PKU diet, current dietary patterns and frequency of consuming high protein foods. The demographics section consisted of questions relating to sex, age, ethnicity, marital status, education and employment status. The questionnaire was piloted with two adults with PKU from the patient panel at the host hospital to test readability, length and clarity.

The questionnaire survey was hosted on an online platform and open for three months between May and August 2021. Five email reminders were sent to patients at the host hospital and advertisements for the survey were reposted multiple times by NSPKU to its members.

### Ethical approval

The study was given favourable ethical opinion by the South West—Central Bristol Research Ethics Committee (REC reference 21/SW/0062; IRAS project ID 291736) and received approval by the Health Research Authority and Health and Care Research Wales. Participant consent was obtained at the start of the online survey. Only respondents who provided consent and completed the full survey were included in the study.

### Statistical analysis

Descriptive statistics were used to report questionnaire responses for continuous data (mean ± SD) and categorical data (*n*, %). Age categories of ≤30 years and >30 years were selected to define two district phases of life that may impact dietary self‐efficacy, and gave sufficiently large numbers in both groups to enable statistical comparison. Perceptions were collapsed into nominal variables as follows: agree (‘strongly agree’, ‘agree’); neutral; and disagree (‘strongly disagree’, ‘disagree’). Paired sample *t* tests were used to compare the dietary regimens advised to participants with that taken by participants, and in comparing knowledge of PKU and of the PKU diet. Associations between knowledge, dietary behaviours and participant characteristics were analysed using Pearson's correlation coefficient for continuous variables, unpaired *t* test for binary variables and one‐way analysis of variance for polychotomous variables with Bonferroni post‐hoc correction. Associations between perceptions, dietary behaviours and participant characteristics were analysed using a chi‐squared or Fisher's exact test, as appropriate. All analyses were conducted using SPSS, version 27.0 (IBM Corp.).

## RESULTS

### Participant characteristics

One hundred and eighty‐nine respondents started the online survey. Nine did not meet eligibility criteria. Of the 180 eligible participants, 43 only partially completed the survey, and were therefore excluded from the analysis. In total, 137 respondents completed the full survey and were included in the study.

Table 1 describes the demographic characteristics of the respondents. Fifty‐seven (41.6%) were male, 78 (56.9%) female and 2 two 1.5%) abstained from specifying their sex. Mean age was 34 years and 4 months (range 16–65 years). Majority were of white ethnicity (97.1%). In terms of formal qualifications, 73 (53.3%) had no university qualifications and 64 (46.7%) had university qualifications. One hundred and three (75.2%) respondents were in full or part‐time employment.

**Table 1:** Respondent characteristics

Subject characteristics (*n* = 137)	*n* (%)
Sex	
Male	57 (41.6)
Female	78 (56.9)
Rather not say	2 (1.5)
Age (years)	
Mean (SD)	34 years and 4 months (10 years and 3 months)
Range	16–65
Age categories	
≤30 years	55 (40.1)
>30 years	82 (59.9)
Ethnicity	
White	133 (97.1)
Asian or Asian British	1 (0.7)
Other ethnic group	1 (0.7)
Rather not say	2 (1.5)
Marital status	
Single	51 (37.2)
Married/civil partnership	53 (38.7)
Co‐habiting	21 (15.3)
Separated/divorced	5 (3.6)
Other^a^	7 (5.1)
Education level	
No university education	73 (53.3)
No formal qualifications	4 (2.9)
School level qualifications	22 (16.1)
Vocational qualifications	20 (14.6)
Advanced School level qualifications	22 (16.1)
Other^b^	5 (3.6)
University education	64 (46.7)
Bachelor's degree (e.g., BSc, BA)	47 (34.3)
Postgraduate degree (e.g., MSc, MA, PhD)	17 (12.4)
Employment status	
Employed (full‐time or part‐time)	103 (75.2)
Not currently employed	34 (24.8)

^a^ The category of ‘Other’ included the following: engaged, in a relationship and not living together and would rather not say.

^b^ The category of ‘Other’ included the following: Higher level teaching assistant status, Certificate of Higher Education, language interpreter and would rather not say.

### Dietary behaviours and metabolic control

Of the 137 respondents, 60 (43.8%) reported following a PKU diet as recommended by their healthcare provider all of their life, 39 (28.5%) had returned to a PKU diet after a period off diet, three (2.2%) returned to a PKU diet specifically for pre‐conception or pregnancy and 35 (25.5%) were not currently on a PKU diet.

Table 2 outlines the dietary behaviours of participants currently following a PKU diet. The prescribed number of PKU exchanges ranged from 2 to 45 (100–2250 mg of phenylalanine) per day (mean ± SD: 11.8 ± 7.4); however, the number of actual PKU exchanges consumed per day was higher at 12.5 ± SD 8.1 (*p* = 0.003). The majority of participants (44; 43.1%) had been prescribed between 5.5 and 10 exchanges (275–500 mg of phenylalanine). Two respondents reported to be advised to consume 45 exchanges per day (2250 mg of phenylalanine). Both reported to be ‘on a PKU diet’ and that their previous phenylalanine concentrations to be within target range. Given the number of exchanges advised, these respondents would likely have a mild PKU as they have a significant protein tolerance. Of the 85 participants who provided data on their PKU exchange regimen, nine (10.6%) reported consuming fewer exchanges than advised, 47 (55.3%) were consuming exchanges as advised and 29 (34.1%) were consuming more than advised.

**Table 2:** Dietary behaviours of respondents currently following a diet for phenylketonuria (PKU) (*n* = 102)

Number of PKU exchanges advised per day^a^	
Mean ± SD	11.8 ± 7.4
Minimum – Maximum	2–45
Exchanges categories, *n* (%)	
≤5 exchanges	12 (11.8)
>5–10	44 (43.1)
>10–15	23 (22.5)
>15–20	10 (9.8)
More than 20 exchanges	7 (6.9)
Don't know	6 (5.9)
Number of PKU exchanges taken per day^a^	
Mean ± SD	12.5 ± 8.1
Minimum – Maximum	2–45
Exchanges categories, *n* (%)	
≤5 exchanges	10 (9.8)
>5–10	34 (33.3)
>10–15	20 (19.6)
>15–20	12 (11.8)
More than 20 exchanges	8 (7.8)
Don't know	18 (17.6)
Exchanges taken vs. advised, *n* (%)	(*n* = 85)
Fewer than advised	9 (10.6)
As advised	47 (55.3)
More than advised	29 (34.1)
Frequency of protein substitute advised per day, *n* (%)	
Once a day	1 (1.0)
Twice a day	8 (7.8)
Three times a day	60 (58.8)
Four or more times a day	26 (25.5)
Don't know	2 (2.0)
Other	5 (4.9)
Frequency of protein substitute taken per day, *n* (%)	
Once a day	5 (4.9)
Twice a day	14 (13.7)
Three times a day	49 (48.0)
Four or more times a day	23 (22.5)
Don't know	0 (0.0)
Other	11 (10.8)
Protein substitute taken vs. advised, *n* (%)	(*n* = 97)
Fewer than advised	16 (16.5)
As advised	77 (79.4)
More than advised	4 (4.1)
Frequency of missing some/all protein substitute, *n* (%)	
Every day	6 (5.9)
Two to four times a week	18 (17.6)
Once a week	19 (18.6)
Once a fortnight	5 (4.9)
Less than once a fortnight	16 (15.7)
I never miss taking my protein substitute	38 (37.3)
Blood phenylalanine target level advised, *n* (%)	
Less than 300 µmol L^–1^	3 (2.9)
Between 120 and 360 µmol L^–1^	9 (8.8)
Between 120 and 600 µmol L^–1^	54 (52.9)
Less than 700 µmol L^–1^	16 (15.7)
Less than 1000 µmol L^–1^	4 (3.9)
I don't have a blood phenylalanine target	2 (2.0)
Don't know	14 (13.7)
Last reported blood phenylalanine level, *n* (%)	
Phenylalanine ≤ 600 µmol L^–1^	52 (51.0)
Less than 120 µmol L^–1^	0 (0)
Between 120 and 360 µmol L^–1^	17 (16.7)
Between 360 and 600 µmol L^–1^	35 (34.3)
Phenylalanine> 600 µmol L^–1^	40 (39.2)
Between 600 and 1000 µmol L^–1^	30 (29.4)
More than 1000 µmol L^–1^	10 (9.8)
Don't know	10 (9.8)

^a^ If range of exchanges reported, value included in analysis was midpoint of the range.

Most respondents (60; 58.8%) were advised to have protein substitutes three times daily. The number of protein substitutes actually taken by respondents (mean ± SD: 3.1 ± 1.1 per day) was significantly lower than the number prescribed (3.3 ± 0.9 per day) (*p* = 0.002). Sixteen (16.5%) were having less protein substitute than advised, 77 (79.4%) were having protein substitutes as advised and 4 (4.1%) were having more than advised. Sixty‐four (68.7%) participants reported not having all supplements, over a third of participants reported not having some or all of their protein substitutes at least once a week, and 5.9% of participants did not have at least one protein substitute daily.

The majority of participants were aiming for a blood phenylalanine of 120–600 µmolL^–1^ (54; 52.9%) or below 700 µmol L^–1^ (16; 15.7%) and 14 (13.7%) did not know their blood phenylalanine target. The most recent phenylalanine level was reported to be <600 µmol L^–1^ by 52 (51%) and >600 µmol L^–1^ by 40 (39.2%) respondents.

Of the 35 respondents currently not following a PKU diet, 23 (65.7%) were following an unrestricted diet, 8 (22.9%) a vegetarian diet, 1 (2.9%) on a vegan diet, and 2 (5.7%) a low protein diet (but not taking any protein substitutes). Over half (18, 51.4%) had been off a PKU diet for more than 10 years.

### Knowledge of PKU and the PKU diet

Table 3 outlines the knowledge questions included in the survey, of which Q1–Q8 related to knowledge of PKU and Q9–Q25 related to knowledge about the PKU diet. The mean ± SD number of correct answers (total knowledge score) was 18.8 ± 3.4 out of 25 (range 5–24), which is 75.2% ± 13.4%. The mean ± SD number of correct answers for knowledge of PKU (PKU knowledge score) was 6.5 ± 1.3 out of 8 (range 1–8), and the mean ± SD number of correct answers for knowledge of the PKU diet (PKU diet knowledge score) was 12.4 ± 2.5 out of 17 (range 4–17). This resulted in a higher mean PKU knowledge score (80.7% ± 16.2%) than PKU diet knowledge score (72.6% ± 14.5%) (*p* < 0.001).

**Table 3:** Respondents' knowledge of phenylketonuria (PKU) and its dietary management

Questions		Correct, *n* (%)	Incorrect, *n* (%)	Don't know, *n* (%)
*Knowledge of PKU*				
Q1	What is the cause of PKU?	133 (97.1)	4 (2.9)	0 (0.0)
Q2	What is the recommended blood phenylalanine level for an adult with PKU in the UK?	97 (70.8)	28 (20.4)	12 (8.8)
Q3	What makes blood phenylalanine levels go high?	132 (96.4)	4 (2.9)	1 (0.7)
Q4	What makes blood phenylalanine levels go too low?	113 (82.5)	17 (12.4)	7 (5.1)
Q5	Blood phenylalanine level is high. What might your dietitian or doctor recommend?	122 (89.1)	12 (8.8)	3 (2.2)
Q6	What will happen to blood phenylalanine level when you fast for 2 days?	71 (51.8)	36 (26.3)	30 (21.9)
Q7	To keep blood phenylalanine level in target range, it is important to do what with your diet?	123 (89.8)	10 (7.3)	4 (2.9)
Q8	Compared to an adult without PKU, an adult with PKU needs how much exercise to keep healthy?	93 (67.9)	28 (20.4)	16 (11.7)
*Knowledge of the PKU diet*				
Q9	What is protein?	131 (95.6)	2 (1.5)	4 (2.9)
Q10	What is phenylalanine?	132 (96.4)	0 (0.0)	5 (3.6)
Q11	When on a PKU diet, the diet should provide how much total protein compared to someone without PKU	2 (1.5)	129 (94.2)	6 (4.4)
Q12	When on a PKU diet, an adult with PKU needs how much energy/calories compared to someone without PKU	85 (62)	40 (29.2)	12 (8.8)
Q13	On a PKU diet, where should most of your protein come from?	132 (96.4)	3 (2.2)	2 (1.5)
Q14	What will happen if someone with PKU is following a low protein diet and not taking their protein substitute daily?	105 (76.6)	24 (17.5)	8 (5.8)
Q15	Why is it necessary to take protein substitutes?	125 (91.2)	6 (4.4)	6 (4.4)
Q16	What is the most important reason for taking your protein substitutes spaced out over the day?	92 (67.2)	41 (29.9)	4 (2.9)
Q17	In general, how much protein is there in one PKU exchange?	111 (81)	8 (5.8)	18 (13.1)
Q18	How many PKU exchanges are in 120 g of broccoli?	56 (40.9)	44 (32.1)	37 (27)
Q19	How many PKU exchanges are in 100 g of tomatoes?	100 (73)	12 (8.8)	25 (18.2)
Q20	How many PKU exchanges are in 180 g of French fries (chips)?	58 (42.3)	37 (27)	42 (30.7)
Q21	What is an exchange‐free food?	83 (60.6)	51 (37.2)	3 (2.2)
Q22	Which product has the most protein per 100 g? (data extraction from food label provided)	128 (93.4)	8 (5.8)	1 (0.7)
Q23	Which product has the most energy/calories per 100 g? (data extraction from food label provided)	133 (97.1)	2 (1.5)	2 (1.5)
Q24	How much protein would you have if you ate a 25 g pack of Product A? (data extraction from food label provided)	128 (93.4)	3 (2.2)	6 (4.4)
Q25	How much of Product B can you have for one PKU exchange? (data extraction and calculation from food label provided)	91 (66.4)	12 (8.8)	34 (24.8)

The majority of questions relating to knowledge of PKU were scored correctly by over 70% of participants (Table 3). By contrast, correct responses relating to knowledge of the PKU diet were more varied. For the question relating to total protein requirements from protein substitutes and PKU exchanges when on a PKU diet (Q11), only two (1.5%) correctly reported needing more than the general population, whereas more than three‐quarters correctly identified the role of protein substitutes in the diet (Q13–Q15). When considering questions related to knowledge of the PKU exchanges in common foods (Q18–Q20), the proportion of participants answering correctly varied from 42.3% to 73%.

Four questions required interpretation of two food package labels (Q22–Q25). Respondents scored highly for questions requiring only data extraction from the food label (>90% correct); however, when required to do calculations using data, 91 (66.4%) provided the correct answer and 34 (24.8%) responded ‘don't know’.

### Knowledge, dietary behaviours and respondent characteristics

Table 4 reports the associations between knowledge scores, PKU dietary behaviours and respondent characteristics. No associations were found between knowledge scores (total knowledge, PKU knowledge, PKU diet knowledge) and sex, age, marital status and employment status. Having a university education was associated with significantly higher knowledge scores compared to those with no university education.

**Table 4:** Associations between knowledge, dietary behaviours and respondent characteristics

	*n*	Total knowledge score (%), mean ± SD	*p* value	PKU knowledge score (%), mean ± SD	*p* value	PKU diet knowledge score (%), mean ± SD	*p* value
Sex							
Male	57	75.0 ± 14.7	0.894	80.7 (16.5	0.981	72.4 ± 15.6	0.866
Female	78	75.3 ± 12.6		80.8 ± 16.1		72.8 ± 13.9	
Age							
≤30 years	55	72.7 ± 15.4	0.076	78.4 ± 16.9	0.184	70.1 ± 16.5	0.087
>30 years	82	76.9 ± 11.8		82.2 ± 15.6		74.4 ± 12.9	
Marital status							
Single	51	72.5 ± 13.6	0.114	78.4 ± 15.0	0.380	69.8 ± 14.9	0.094
Married/civil partnership	53	78.3 ± 13.8		84.0 ± 17.1		75.7 ± 14.6	
Co‐habiting	21	76.6 ± 11.6		80.4 ± 16.1		74.8 ± 12.2	
Separated/divorced	5	66.4 ± 6.1		77.5 ± 10.5		61.2 ± 10.7	
Other	7	73.1 ± 14.4		75.0 ± 20.4		72.3 ± 15.5	
Education level							
No university education	73	71.6 ± 14.1	< 0.001	76.5 ± 18.2	0.001	69.3 ± 15.1	0.004
University education	64	79.3 ± 11.4		85.4 ± 12.1		76.5 ± 12.9	
Employment status							
Employed (full time or part‐time)	103	75.8 ± 13.0	0.339	81.8 ± 15.8	0.152	73.0 ± 14.1	0.582
Not currently employed	34	73.3 ± 14.8		77.2 ± 17.0		71.5 ± 16.1	
PKU diet pattern							
Always been on diet	60	78.0 ± 12.0^a^	0.006	84.0 ± 12.8^a^	0.042	75.2 ± 14.3^a^	0.009
Returned to diet	42	76.3 ± 12.3^a,b^		80.4 ± 16.6^a,b^		74.4 ± 12.4^a^	
Off diet	35	69.1 ± 15.4^b^		75.4 ± 19.5^b^		66.2 ± 15.7^b^	
PKU exchanges advised	96	PCC = 0.054	0.601	PCC = 0.107	0.300	PCC = 0.016	0.875
PKU exchanges consumed	84	PCC = 0.101	0.359	PCC = 0.097	0.381	PCC = 0.82	0.457
PKU exchanges adherence							
Fewer than advised	9	74.2 ± 16.0	0.106	86.1 ± 13.2	0.673	68.6 ± 20.4^a^	0.035
As advised	47	77.8 ± 11.3		83.2 ± 14.3		75.2 ± 12.5^a,b^	
More than advised	29	82.1 ± 8.0		85.8 ± 12.0		80.3 ± 8.2^b^	
Protein substitute advised	98	PCC = −0.029	0.780	PCC = −0.072	0.480	PCC = −0.001	0.988
Protein substitute consumed	98	PCC = 0.103	0.313	PCC = 0.026	0.801	PCC = 0.121	0.236
Protein substitute adherence							
Fewer than advised	16	72.3 ± 17.3	0.088	78.1 ± 22.1	0.125	69.5 ± 17.9	0.156
As advised	77	79.2 ± 10.3		84.6 ± 11.5		76.6 ± 12.3	
More than advised	4	74.0 ± 13.7		75.0 ± 17.7		73.5 ± 15.6	
Last reported phenylalanine level							
Between 120 and 360 µmol/L	17	80.0 ± 9.9	0.096	86.0 ± 12.4	0.061	77.2 ± 11.9	0.199
Between 360 and 600 µmol L^–1^	35	78.5 ± 12.2		85.7 ± 11.8		75.1 ± 14.9	
Between 600 and 1000 µmol L^–1^	30	79.6 ± 11.4		82.9 ± 14.1		78.0 ± 12.4	
More than 1000 µmol L^–1^	10	69.2 ± 15.2		72.5 ± 21.9		67.7 ± 13.7	
Years since discontinuing diet							
Less than 3 years ago	12	73.3 ± 10.1	0.409	79.2 ± 11.1	0.626	70.6 ± 12.8	0.397
3–10 years ago	5	71.2 ± 15.9		77.5 ± 16.3		68.2 ± 16.4	
More than 10 years ago	18	65.8 ± 17.9		72.2 ± 24.5		62.7 ± 17.1	

*Note*: *p* values comparing two groups are the result of independent *t* tests (two groups). *p* values comparing three groups are the result of an analysis of variance, when a statistically significant difference was identified across the three groups, comparisons were made between two groups using a Bonferroni post hoc correction, in which case values that do not share a common superscript letter are statistically significantly different.

Abbreviations: PCC, Pearson's correlation co‐efficient and *p* value or the association; PKU, phenylketonuria.

There were significant associations between knowledge scores (total knowledge, PKU knowledge, PKU diet knowledge) and whether a respondent always followed a PKU diet, had returned to diet or was currently off diet. Post‐hoc analysis showed significantly lower correct scores amongst those who are currently off diet (mean ± SD total knowledge score 69.1% ± 15.4%) compared to those who have always followed their recommended PKU diet (78.0% ± 12.0%, *p* = 0.005). No significant differences were found in knowledge scores of respondents who have returned to a PKU diet (mean ± SD total knowledge score 76.3% ± 12.3%) and those who have always followed a PKU diet (78.0% ± 12.0%, *p* = 1.00). Knowledge scores of respondents who have returned to a PKU diet were higher than those who were off diet, but findings only reach statistical significance for PKU diet knowledge scores (74.4% ± 12.4% vs. 66.2% ± 15.7%, *p* = 0.039).

No associations were found between knowledge scores and either the number of PKU exchanges (advised or taken) or the number of protein substitutes (advised or taken). PKU diet knowledge scores were higher in those consuming more exchanges than advised compared to those consuming fewer than advised. Knowledge scores were not associated with taking less, more or same protein substitute than advised.

A trend towards lower knowledge scores in those with higher phenylalanine levels (>1000 µmol L^–1^) was observed, but this association was not significant (*p* = 0.096). For respondents who were currently off diet, there was no significant association between knowledges scores and years since discontinuing a PKU diet.

### Perception of the PKU diet

Table 5 outlines respondents' perceptions of the PKU diet. Majority agreed that when following a PKU diet, the diet will keep them well (114; 87%), of whom 56/60 (93.3%) had always followed a PKU diet, 37/42 (88.1%) had returned to diet and 21/35 (60.0%) were ‘off diet’. Therefore, more than half of participants who were off diet also believed that being on a PKU diet will keep them well.

**Table 5:** Respondents' perceptions of the phenylketonuria (PKU) diet

			Responses, *n* (% of *N*)				
	Don't know, *n* (%)^b^	Valid response, *N* =	Strongly agree	Agree	Neither agree or disagree	Disagree	Strongly disagree
The PKU diet I am recommended to follow:							
Is healthy	8 (5.8)	129	35 (27.1)	57 (44.2)	18 (14.0)	12 (9.3)	7 (5.4)
Gives me all the vitamins and minerals I need	5 (3.6)	132	49 (37.1)	66 (50.0)	5 (3.8)	11 (8.3)	1 (0.8)
Gives me all the energy/calories I need	5 (3.6)	132	29 (22.0)	72 (54.5)	15 (11.4)	12 (9.1)	4 (3.0)
Gives my body enough protein	5 (3.6)	132	31 (23.5)	84 (63.6)	13 (9.8)	3 (2.3)	1 (0.8)
Is higher in sugar compared to the diet for the general population	14 (10.2)	123	32 (26.0)	48 (39.0)	25 (20.3)	15 (12.2)	3 (2.4)
Is higher in carbohydrates compared to the diet for the general population	15 (10.9)	122	38 (31.1)	55 (45.1)	16 (13.1)	10 (8.2)	3 (2.5)
Is higher in fats compared to the diet for the general population	15 (10.9)	122	14 (11.5)	36 (29.5)	39 (32.0)	31 (25.4)	2 (1.6)
Is higher in fruits and vegetables compared to the diet for the general population	9 (6.6)	128	47 (36.7)	60 (46.9)	14 (10.9)	6 (4.7)	1 (0.8)
When I am following my recommended PKU diet:							
My protein substitutes need to be taken daily	3 (2.2)	134	102 (76.1)	28 (20.9)	3 (2.2)	0 (0.0)	1 (0.7)
Taking my protein substitutes daily is not as important as following a low protein diet for PKU	5 (3.6)	132	1 (0.8)	8 (6.1)	15 (11.4)	54 (40.9)	54 (40.9)
I don't understand why I need to take protein substitutes	3 (2.2)	134	1 (0.7)	3 (2.2)	9 (6.7)	36 (26.9)	85 (63.4)
The number of protein substitutes I have been recommended to take a day was right for me	11 (8.0)	126	22 (1.5)	73 (57.9)	17 (13.5)	11 (8.7)	3 (2.4)
I get concerned that the amount of protein substitutes that I have been recommended to take is too many	6 (4.4)	131	3 (2.3)	3 (2.3)	23 (17.6)	64 (48.9)	38 (29.0)
I get concerned that the amount of protein substitutes that I have been recommended to take is too few	7 (5.1)	130	7 (5.4)	12 (9.2)	19 (14.6)	63 (48.5)	29 (22.3)
Taking my protein substitutes, I feel confident that I am getting enough protein	8 (5.8)	129	27 (20.9)	76 (58.9)	14 (10.9)	9 (7.0)	3 (2.3)
My protein substitutes help to improve my blood phenylalanine levels	9 (6.6)	128	41 (32.0)	64 (50.0)	20 (15.6)	2 (1.6)	1 (0.8)
When I am following my recommended PKU diet:							
I feel that the diet will keep me well	6 (4.4)	131	35 (26.7)	79 (60.3)	9 (6.9)	5 (3.8)	3 (2.3)
I get concerned about not having enough protein to keep healthy	4 (2.9)	133	6 (4.5)	21 (15.8)	22 (16.5)	58 (43.6)	26 (19.5)
I get concerned about not having enough protein to build muscle	6 (4.4)	131	17 (13.0)	27 (20.6)	22 (16.8)	46 (35.1)	19 (14.5)
I get concerned about my long‐term health	4 (2.9)	133	36 (27.1)	35 (26.3)	21 (15.8)	30 (22.6)	11 (8.3)
I feel well when I am not on the PKU diet	19 (13.9)	118	10 (8.5)	20 (16.9)	20 (16.9)	25 (21.2)	43 (36.4)
When someone with PKU exercises:							
The PKU diet will give them all the vitamins and minerals they need	16 (11.7)	121	22 (18.2)	63 (52.1)	20 (16.5)	15 (12.4)	1 (0.8)
The PKU diet will give them all the protein they need	15 (10.9)	122	20 (16.4)	54 (44.3)	18 (14.8)	24 (19.7)	6 (4.9)
The PKU diet will give them all the carbohydrates they need	19 (13.9)	118	29 (24.6)	66 (55.9)	18 (15.3)	5 (4.2)	0 (0.0)
The PKU diet will give them all the energy/calories they need	18 (13.1)	119	21 (17.6)	62 (52.1)	14 (11.8)	20 (16.8)	2 (1.7)

^b^ % of responses out of the total responses (*n* = 137). These responses are excluded from the data analysis.

Greater variability in respondents' perceptions, with responses distributed across the agree, neutral and disagree, were seen for statements relating to the PKU diet's fat content, provision of adequate protein to keep healthy and to build muscle including during exercise, and impact on long‐term health. Furthermore, perception varied for how someone felt when not on the PKU diet (Table 5).

### Perception of the PKU diet, dietary behaviours and respondent characteristics

The majority of participants' perceptions of the PKU diet were not associated with dietary behaviours, and no associations were found between perceptions and participant characteristics (data not shown). Table 6 outlines perceptions and dietary behaviours found to be associated. Participants' perceptions of the following statements were found to be significantly associated with whether they always followed a PKU diet, returned to diet or were currently off diet: ‘when on a PKU diet, I get concerned about my long‐term health’, ‘I feel that the diet will keep me well’ and ‘I feel well when I am not on the PKU diet’.

**Table 6:** Associations between perceptions of the Phenylketonuria (PKU) diet, dietary behaviours and respondent characteristics

	Agree, *n* (%)	Neutral, *n* (%)	Disagree, *n* (%)	*p* value
When on a PKU diet, I get concerned about my long‐term health				
PKU diet pattern				
Always on diet	31 (43.7)	10 (47.6)	19 (46.3)	0.009
Returned to diet	30 (42.3)	2 (9.5)	10 (24.4)	
Off diet	10 (14.1)	9 (42.9)	12 (29.3)	
Protein substitute adherence				
Taking fewer than advised	11 (19.6)	4 (33.3)	1 (3.4)	0.049
Taking as advised	43 (76.8)	7 (58.3)	27 (93.1)	
Taking more than advised	2 (3.6)	1 (8.3)	1 (3.4)	
I feel well when I am not on the PKU diet				
PKU diet pattern				
Always on diet	4 (13.3)	6 (30.0)	35 (51.5)	< 0.001
Returned to diet	7 (23.3)	11 (55.0)	24 (35.3)	
Off diet	19 (63.3)	3 (15.0)	9 (13.2)	
I feel that the diet will keep me well				
PKU diet pattern				
Always on diet	56 (49.1)	1 (11.1)	2 (25.0)	0.011
Returned to diet	37 (32.5)	2 (22.2)	3 (37.5)	
Off diet	21 (18.4)	6 (66.7)	3 (37.5)	
When someone with PKU exercises, the PKU diet will give them all the protein they need				
PKU exchange adherence				
Consuming fewer than advised	6 (13.0)	3 (27.3)	0 (0.0)	0.014
Consuming as advised	27 (58.7)	7 (63.6)	8 (40.0)	
Consuming more than advised	13 (28.3)	1 (9.1)	12 (60.0)	

Of the participants currently following a PKU diet, 61 (86%) remained concerned about their long‐term health when on diet, whereas 29 (70.7%) did not have concerns for their long‐term health. Of the 35 participants currently off diet, 19 (63.3%) felt well when not on the diet and 9 (13.2%) did not feel well off diet.

A participant's perception of whether the PKU diet provides adequate protein when exercising was associated with adherence to prescribed PKU exchanges, whereas those who ‘disagreed’ were predominately taking more exchanges than advised (Table 6).

Of note, perceptions of the following statements were not associated with adherence to PKU exchanges or protein substitutes: ‘when on a PKU diet, I get concerned about not having enough protein to keep healthy’ and ‘when on a PKU diet, I get concerned about not having enough protein to build muscle’.

### Importance of research in the dietary management and health of adults with PKU

The survey included questions to understand how important two research areas were for adults with PKU. Research to understand the impact of different amounts, frequency and/or types of protein substitutes on muscle and general health was seen as extremely or very important by majority of respondents (104; 75.9%). Research to understand how protein substitutes can be tailored (amount, frequency and/or type) to match personal muscle and personal activity levels was also seen as extremely or very important by majority of respondents (99; 72.3%).

## DISCUSSION

This is the first nationwide study investigating both knowledge and perceptions of PKU and its dietary management, and the associations with dietary behaviours in a large cohort of adults with PKU.

Overall, respondents demonstrated good knowledge of both PKU in general and the PKU diet; however, knowledge of the former was greater than knowledge of the latter. Incorrect or ‘don't know’ responses were more frequent in questions related to PKU exchanges of common foods, a finding shown in previous studies,^3^, ^6^, ^8^ and the question requiring data extraction and calculations from food labels. Retention of knowledge regarding PKU exchanges, as well as the ability to determine PKU exchanges from food labels, requires cognitive skills such as memory, attention and information processing. Deficits in cognitive functioning have been reported, even amongst those adhering to their phenylalanine restricted diet.^12^ This present study highlights that even patients currently on a PKU diet, and likely in regular contact with dietitians, may experience some cognitive dysfunction that impacts label reading and diet recall and they may benefit from more structured refresher education sessions at regular intervals.

Respondent characteristics were not predictors of knowledge, with the exception of having a university education, which inevitably may be associated with improved knowledge acquisition and utilisation. As expected, those who have always followed a PKU diet had greater knowledge of PKU and its dietary management. Interestingly, no difference in knowledge was found between participants who have returned to diet and those who have always followed a diet, demonstrating that either (i) adults returning to diet can gain equivalent levels of knowledge to those who have always been on diet or (ii) that higher knowledge of PKU and diet was associated with the decision to return to diet. Participants who had returned to diet, had greater knowledge of the PKU diet than participants off diet. It is unclear whether knowledge was the catalyst to returning to diet or whether as part of the return to diet, participants received increased education and training on the PKU diet.

Knowledge did not predict adherence to using protein substitutes or consuming PKU exchanges as advised. Although not significant, lower knowledge scores were associated with poorer metabolic control, findings which have been reported when considering caregivers' knowledge and their children's metabolic control.^5^, ^8^

To support dietary adherence for adults with PKU, establishing an individual's baseline knowledge of PKU and the PKU diet is important to identify their further education and training needs. Routine clinic appointments may not allow adequate time for assessment of knowledge and provision of further training and education, and therefore establishing additional clinics dedicated to enhancing knowledge of PKU and the dietary management may be warranted. Written resources are typically provided with dietary management information^4^; however, with consideration for different learning styles, training sessions could be extended to include short educational videos, use of mobile applications and hands on practical sessions such as working through mathematic calculations to determine PKU exchanges using different food labels.

The majority of participants shared the perception that the PKU diet will keep them well, a positive perception that was also shown in a previous study where majority of adults with PKU reported to feel better when on a PKU diet.^9^ However, participants in the present study shared similar perceptions of the PKU diet whether they were currently off diet, had a period off the PKU diet or always followed a PKU diet. In this regard, 21 (60.0%) participants off diet shared the belief that following a PKU diet will keep them well. Given this significant number, further research to assess barriers to being on diet and evaluate methods to overcome these barriers is important.

Perceptions of the PKU diet did not appear to predict dietary behaviours, with the exception of a participant's perception of whether they have concerns for their long‐term health when on a PKU diet and whether they feel well when not following a PKU diet. Lifelong treatment is recommended as the long‐term health outcomes of high phenylalanine levels is currently unknown.^2^ However, even amongst participants who have always followed a diet, concerns remain about their long‐term health. This highlights the importance of further research in adults and older adults with PKU into understanding the long‐term neurocognitive, physical and functional outcomes. Although the long‐term outcomes are unknown, for many participants, feeling well when on the PKU diet will be the motivator for remaining on diet. This was shown in the current study where participants who disagreed with the statement regarding feeling well when not following a PKU diet were predominately on diet; however, 13.2% of participants who were off diet also disagreed with this statement, demonstrating the complexities of factors influencing adherence to a PKU diet.

Disagreeing that the PKU diet provides all the protein needed to exercise was associated with taking more exchanges than advised. Limited research has focused on the impact of exercise on protein requirements in PKU, and the present study has highlighted concerns that exist amongst adults with PKU. Furthermore, research to understand how protein substitutes can be tailored to match body composition and physical activity levels was ranked by majority of respondents as extremely or very important. Further research is needed to explore how these factors influence dietary requirements in adults with PKU.

### Study strengths and limitations

The strengths of the present study include the recruitment method leading to one of the largest ever samples of adults with PKU, and from across the whole of the UK, together with the novel use of a comprehensive assessment for perceptions of the PKU diet. Moreover, including participants both on and off a PKU diet allowed insight to be gained on the influence of knowledge and perceptions on dietary adherence. However, a higher proportion of participants were currently following a PKU diet compared to reports in previous UK based studies,^1^, ^4^ despite adherence to protein substitutes being similar^1^, ^4^; meanwhile, a high proportion of respondents had university education (46.7%) compared to the general UK population (27% with level 4 or above).^13^ Therefore, selection bias might have encouraged more knowledgeable patients to participate and thus the knowledge of the general PKU population may indeed be lower than measured here. It should be noted that knowledge about PKU and the diet may encompass more than the 25 questions included in the questionnaire; however, the questionnaire used had the advantage of including questions on the different aspects of dietary management.

## CONCLUSIONS

It is well established that adherence to a PKU diet reduces with age, and therefore gaining an understanding of the factors that influence dietary adherence is essential to removing barriers to improving dietary management and patient outcomes for adults with PKU. The present study found knowledge to be associated with dietary adherence. Ongoing dietetic input is needed to further enhance knowledge of the PKU diet and in developing skills to determine and calculate PKU exchanges of foods, which are essential tools for successful dietary management. It is of interest that participants shared similar perceptions of the diet, despite their history with adherence to their PKU diet, an area that requires further research to understand the motivators and beliefs that influence dietary adherence.

## AUTHOR CONTRIBUTIONS

Sarah J. Firman, Radha Ramachandran and Kevin Whelan were involved in the conceptualisation and design of the questionnaire survey. Sarah J. Firman led on recruitment and data analysis, with support from Kevin Whelan who provided supervision. The original draft was prepared by Sarah J. Firman. Radha Ramachandran and Kevin Whelan provided critical revision of the draft. Sarah J. Firman, Radha Ramachandran and Kevin Whelan revised and approved the final version of the manuscript submitted for publication.

## CONFLICTS OF INTEREST

Sarah J. Firman has received funding to attend conferences and study days from Nutricia, Vitaflo International and Dr Schär UK Ltd, as well as consulting fees from Vitaflo International and Meta Healthcare Ltd. Kevin Whelan is in receipt of research funding from Danone and has acted as a consultant for Danone.

## ETHICAL STATEMENT

The study was given favourable ethical opinion by the South West—Central Bristol Research Ethics Committee (REC reference 21/SW/0062; IRAS project ID 291736) and received approval by the Health Research Authority and Health and Care Research Wales.

## TRANSPARENCY DECLARATION

The lead author affirms that this manuscript is an honest, accurate and transparent account of the study being reported. The reporting of this work is compliant with STROBE guidelines. The lead author affirms that no important aspects of the study have been omitted and that any discrepancies from the study as planned have been explained (REC reference 21/SW/0062; IRAS project ID 291736).
